# Clinical and Angiographic Predictors of Acute Kidney Injury in ST-Elevation Myocardial Infarction Treated With Primary Percutaneous Coronary Intervention

**DOI:** 10.7759/cureus.96052

**Published:** 2025-11-03

**Authors:** Imran Khan, Malik Faisal Iftekhar, Zaland A Yousafzai, Qazi Kamran Amin, Ihtesham Shafiq

**Affiliations:** 1 Interventional Cardiology/Electrophysiology, Lady Reading Hospital Medical Teaching Institution, Peshawar, PAK; 2 Cardiology/Electrophysiology, Lady Reading Hospital Medical Teaching Institution, Peshawar, PAK; 3 Medicine, Rehman Medical Institute, Peshawar, PAK; 4 Medical Research, Rehman Medical Institute, Peshawar, PAK; 5 Internal Medicine, Rehman Medical Institute, Peshawar, PAK

**Keywords:** acute kidney injury, contrast-induced nephropathy, percutaneous coronary intervention, primary pci, stemi

## Abstract

Background

Acute kidney injury (AKI) is a significant consequence of primary percutaneous coronary intervention (PCI) for ST-segment elevation myocardial infarction (STEMI). It is linked to greater mortality, longer hospital stays, and increased morbidity. Improving healthcare outcomes requires identifying individuals who are at high risk.

Objective

The aim of this study is to ascertain the prevalence, predictors, and immediate consequences of AKI in STEMI patients receiving initial PCI.

Methodology

From January 2023 to December 2024, 207 adult STEMI patients who received primary PCI at a tertiary cardiac center were included in this prospective observational study. Kidney Disease: Improving Global Outcomes (KDIGO) criteria were used to define AKI. Angiographic, clinical, and baseline demographic data were documented. To find independent predictors of AKI, multivariate logistic regression analysis was employed, and discriminative power was evaluated using receiver operating characteristic (ROC) curves.

Results

AKI occurred in 56 patients (27.05%). Diabetes, hypertension, age, and gender did not significantly differ between the AKI and non-AKI groups. However, AKI patients experienced more clinical shock, anterior wall myocardial infarction, left anterior descending (LAD) lesions, a higher Killip class, and a substantially longer symptom-to-door time. Clinical shock, LAD lesion, contrast volume >145 ml, and Killip class III/IV were independent predictors of AKI. The highest ROC area under curve (AUC) was observed for Killip class III/IV (AUC=0.81).

Conclusion

Clinical shock, LAD involvement, a large contrast volume, and Killip class III/IV are significant predictive factors for AKI following PCI. Prevention requires early risk categorization and contrast-minimizing approaches.

## Introduction

Acute kidney injury (AKI) is linked to both short- and long-term negative consequences in patients receiving primary angioplasty for acute ST-elevation myocardial infarction (STEMI) [1]. In STEMI patients undergoing reperfusion therapy with primary angioplasty, the development of AKI has a substantial impact on morbidity and mortality, highlighting its clinical significance. Therefore, in order to improve both the short-term and long-term outcomes in this high-risk population, preventive strategies to reduce the incidence of AKI during the procedure are crucial [2]. The incidence rate of AKI varies between 7.2% and over 20% among patients who receive primary percutaneous coronary intervention (PCI) [3,4]. These figures demonstrate the complex nature of AKI that depends on patient characteristics, clinical scenarios, and the diagnostic criteria used.

Beyond its immediate complications, acute renal injury after primary angioplasty is a cause for concern because it is linked to negative in-hospital outcomes, such as extended hospital stays and a higher chance of developing chronic kidney disease (CKD) [5,6]. Diabetes influences multiple risk factors for AKI and is a major factor, as these patients frequently demonstrate underlying microvascular dysfunction and an increased vulnerability to contrast-induced nephropathy. Furthermore, those with diabetes who have STEMI are more likely to have several comorbidities and concurrent hemodynamic instability, which makes them more vulnerable to AKI [7,8].

According to recent research, wide pulse pressure and an elevated glycemic gap (the difference between admission glucose levels and anticipated average glucose), are two prominent indicators of AKI in patients with diabetes undergoing primary angioplasty [9]. In this high-risk group, pharmacological approaches, in particular the use of sodium-glucose cotransporter 2 inhibitors (SGLT2is), have also shown promise in lowering the risk of contrast-induced nephropathy [10-12]. However, the prevalence of AKI in the diabetic population continues to be a significant clinical challenge in spite of improvements in risk assessment and the implementation of preventive measures [8,13].

The rationale for this study was to assess the burden and prognostic implications of AKI in the context of primary PCI. To maximize patient outcomes, early detection of high-risk patients may allow for efficient risk categorization and well-informed decision-making. The expected results could help design focused preventive measures, like pharmacological protection, proper hydration, and contrast minimization, which would ultimately lower morbidity, mortality, and medical expenses. Additionally, this study attempts to improve periprocedural management practices in STEMI patients and inform clinical guidelines, which will raise the standard of cardiac care delivery.

## Materials and methods

This prospective observational study was carried out from January 2023 to December 2024 at Lady Reading Hospital in Peshawar, Pakistan. The Institutional Review Board at Lady Reading Hospital, Peshawar, gave its approval to the study (ref no. 1321/LRH/MTI). Written informed permission was acquired from every participant.

The sample size was identified to have sufficient power in finding potentially significant changes between the outcomes of patients with and without AKI, and eventually increase the dependability of our results.

Sample size justification

To assume adequate power (>80%) to identify statistically significant outcomes between the AKI and non-AKI groups, the sample size of 207 patients was computed on the basis of the anticipated incidence of AKI following a PCI (reported in the literature as 16% with a 95% level of confidence and a 5% percent error limit) [3].

Inclusion and exclusion criteria

All adult patients (≥18 years) with a STEMI diagnosis who received primary PCI within 12 hours of symptom onset were eligible. Exclusion criteria included chronic dialysis, recent contrast exposure (within seven days), hemodynamic instability requiring emergency surgery, incomplete or deleted data, and chronic kidney disease stage 4/5 (estimated glomerular filtration rate or eGFR <30 mL/min/1.73 m²).

Data collection

The collected data encompassed demographics, medical history, laboratory values (serum creatinine, hemoglobin, uric acid, eGFR), clinical parameters (Killip class, blood pressure, heart rate), and angiographic findings (culprit lesion, contrast volume, TIMI flow). According to KDIGO criteria, AKI is defined as an increase in serum creatinine of ≥0.3 mg/dL within 48 hours or an increase of ≥1.5 times the baseline within seven days [14].

Data analysis

IBM SPSS Statistics for Windows, Version 25 (Released 2017; IBM Corp., Armonk, New York, United States) was used to conduct the statistical analysis. Mean ± SD was used to represent continuous variables, and N (%) was used to represent categorical variables. Groups were compared using t-tests and chi-squared. Using multivariate logistic regression, independent predictors of AKI were found. Discriminative power was evaluated using ROC curves.

## Results

Baseline characteristics and clinical presentation

Among the 207 patients in whom PCI was performed due to STEMI, AKI was recorded in 27.05% (n=56) cases. The cohort was composed of 80.1% (n=166) male patients and 19.8% (n=41) female patients with a mean age of 56 +/- 11 years. Patients with AKI got considerably greater contrast volumes (155.66 ± 73.12 vs. 95.88 ± 74.32 mL, p<0.001) and had significantly longer symptom-to-door duration (197 ± 45 vs. 113 ± 23 minutes, p=0.030), compared to those without AKI. Age, blood pressure, heart rate, hemoglobin, creatinine, eGFR, uric acid, and random blood sugar levels did not significantly differ across the groups. The AKI group demonstrated an elevated serum uric acid (7.76 mg/dL + 3.88) level, and hemoglobin was reduced. These variables were strongly associated with post-PCI AKI and should be considered for early risk stratification. The levels of serum creatinine and eGFR did not differ significantly at baseline. The continuous variables have been tabulated in Table 1.

**Table 1:** Clinical and laboratory parameters of patients with and without AKI following PCI eGFR: Estimated glomerular filtration rate; AKI: Acute kidney injury; PCI: Percutaneous coronary intervention.

Variables	AKI (n=56)		No AKI (n=151)		P value
	Mean	Standard deviation	Mean	Standard deviation	
Age (years)	59	11.9	60	10.6	0.538
Average symptom time (minutes)	197	45	113	23	0.03
Average door-to-balloon time (minutes)	30	11	22	13	0.7
Systolic blood pressure (mmHg)	124.29	26.02	124.87	35.88	0.7
Diastolic Blood Pressure (mmHg)	75.5	16.5	77.66	14.84	0.38
Heart rate (bpm)	85	26	85	27	0.117
Hemoglobin (g/dL)	12.48	6.99	13.56	7.33	>0.05
Creatinine (mg/dL)	0.8	0.21	0.91	0.34	>0.05
eGFR (mL/min/1.73m^2^)	74.54	36.66	71.54	33.66	>0.05
Uric acid (mg/dL)	7.76	3.88	6.69	3.77	>0.05
Random blood sugar (mg/dL)	177.87	88	216.68	141.53	>0.05
Total contrast volume (mL)	155.66	73.12	95.88	74.32	<0.001

The prevalence of diabetes mellitus and dyslipidemia was comparable in the two groups. Diabetes was present in 39.3% of patients with AKI and 39.1% of those without AKI , while dyslipidemia was observed in 30.4% and 30.5% of patients in AKI and non-AKI groups respectively (p>0.05). Additionally, there was no significant difference in the proportion of patients with hypertension (64.3% vs. 68.9%), current smokers (55.4% vs. 55.6%), or the distribution of male patients (80.4% vs. 80.1%) between the AKI and non-AKI groups. However, advanced Killip class III/IV (69.6% vs. 10.6%, p<0.001) and clinical shock (50.0% vs. 6.6%, p<0.001) were more common in patients with AKI compared to those without AKI upon admission, respectively. LAD was the main culprit vessel (69.6% vs. 28.3%, p<0.001), and anterior wall myocardial infarction was substantially more common in the AKI vs. non-AKI group (30.4% vs. 25.8%, p=0.001; Table 2).

**Table 2:** Baseline categorical variables in the patient population AKI: Acute Kidney Injury, MI: Myocardial Infarction; LAD: Left Anterior Descending.

Variable	Category	AKI (n=56)		No AKI (n=151)		P-value
		Count	Percentage	Count	Percentage	
Gender	Male	45	80.4	121	80.1	0.18
Gender	Female	11	19.6	30	19.9	0.11
Hypertension	Yes	36	64.3	104	68.9	0.367
Diabetes	Yes	22	39.3	59	39.1	0.345
Dyslipidemia	Yes	17	30.4	46	30.5	0.932
Current smoking	Yes	31	55.4	84	55.6	0.345
Killip class	III/IV (combined)	39	69.6	16	10.6	<0.001
Clinical shock	Yes	28	50	10	6.6	<0.001
MI location	Anterior MI	17	30.4	39	25.8	<0.001
Culprit vessel	LAD	39	69.6	43	28.5	<0.001

The main discovery was the higher contrast volume at the PCI of the patients who developed AKI (155.66 +/- 73.12 ml vs. 95.88 +/- 74.32 mL; p=0.001). This and other significant findings have been further stratified using regression analysis and were identified as independent predictors of AKI. These were strongly associated with post-PCI AKI and should be considered for early risk stratification (Table 3).

**Table 3:** Independent predictors of AKI LAD: Left Anterior Descending; AKI: Acute Kidney Injury.

Variable	Odds Ratio	95 % Confidence Interval	P value
Killip Class III/IV	13.85	7.43-25.82	<0.0001
Contrast volume	11.38	6.07-21.32	<0.0001
LAD lesion	4.88	2.67-8.92	<0.0001
Clinical shock	3.27	1.85-5.77	0.0001

**Figure 1:**
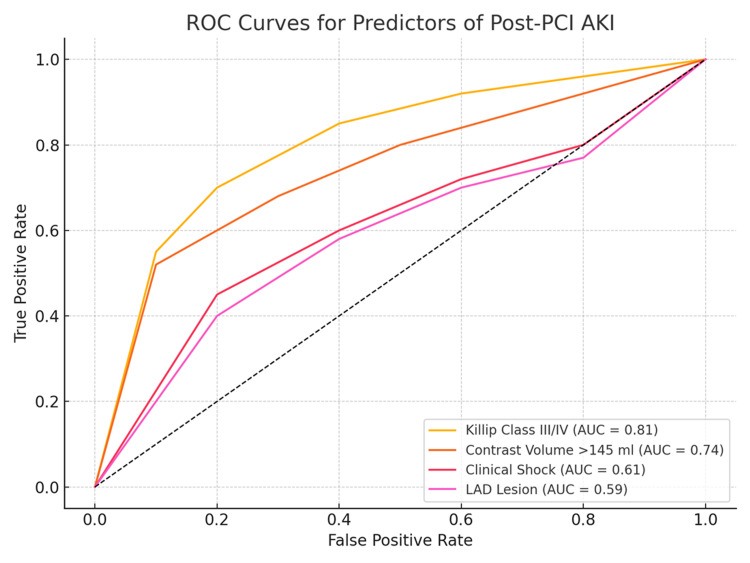
ROC Curve Analysis AUC: Area Under Curve; ROC: Receiving Operating Curve; LAD: Left Anterior Descending.

To undertake further assessment of the discriminative power of relevant the predictors, the analysis of ROC curve was carried out. Figure 1 shows the ROC curves for the nine best AKI predictors.

The greatest predictive powers pertained to the Killip class III/IV (AUC=0.81), which was closely followed by contrast volume (AUC=0.74).

**Figure 2:**
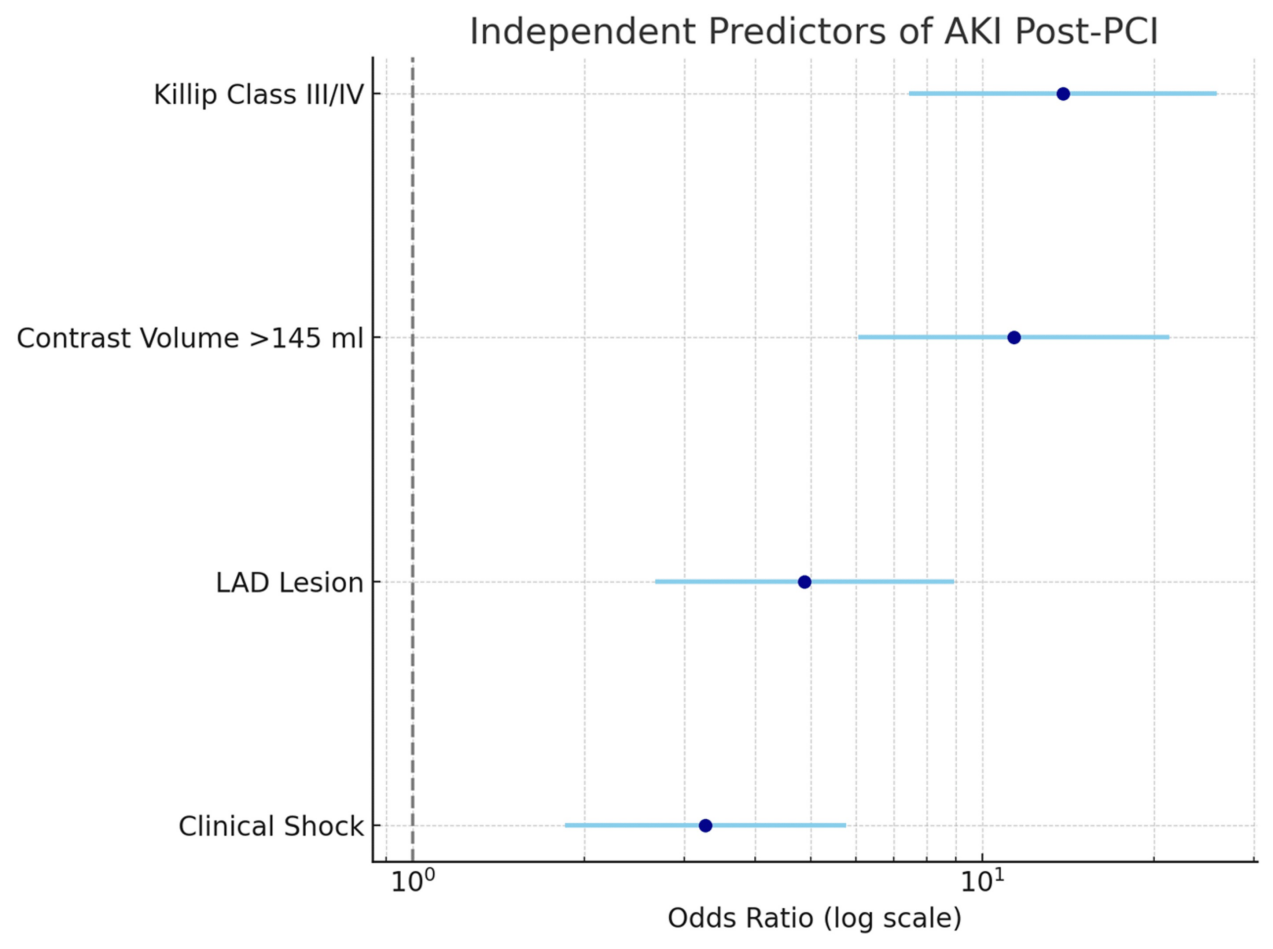
Forest plot visually representing the independent predictors of AKI following PCI AKI: Acute Kidney Injury; PCI: Percutaneous Coronary Intervention.

Figure 2 demonstrates that AKI following PCI can be independently predicted by Killip class III/IV, contrast volume >145 ml, LAD damage, and clinical shock.

The strongest risk factors were Killip class III/IV and high contrast volume; significant but relatively weaker relationships were seen between clinical shock and LAD damage.

## Discussion

The current paper demonstrated an AKI prevalence of 27% in STEMI patients receiving primary PCI, consistent with previous reports indicating an incidence range between 10% and 30%, depending on the population characteristics and AKI definitions employed [15,16]. This aligns with findings from a large retrospective cohort of 2,912 patients with STEMI, where AKI incidence was 7.6%, with early AKI strongly associated with cardiogenic shock and worse outcomes, underscoring the impact of hemodynamic instability on renal injury [15]. Similarly, studies have repeatedly identified Killip class III/IV and clinical shock as the strongest non-modifiable predictors of AKI, reflecting the pathophysiological role of reduced cardiac output impairing renal perfusion and causing ischemic renal injury [15,17].

A notable modifiable risk factor identified was contrast volume, with patients receiving >145 ml at significantly higher risk. This finding is consistent with multiple investigations, emphasizing contrast-induced nephropathy (CIN) as a key contributor to AKI during PCI [18,19]. For example, a study analyzing 2,011 patients with STEMI found contrast volume to be a critical determinant of AKI, advocating for contrast minimization and pre-procedural hydration as standard care, especially in high-risk groups [17]. However, some recent data suggest that the absolute contrast volume threshold may vary based on baseline renal function and other comorbidities, indicating the need for individualized risk assessment [20]. Other important predictors included anterior myocardial infarction and LAD involvement. These infarcts tend to involve larger myocardial territories, provoking more profound hemodynamic compromise and systemic inflammatory responses, both of which can have a detrimental impact on renal function [21]. This is supported by findings from the Bremen STEMI Registry, where higher AKI stages correlated with greater infarct size and worse hemodynamics [15]. Conversely, a few studies have reported less pronounced associations between infarct location and AKI, suggesting that other factors, such as microvascular obstruction and reperfusion injury, may modulate risk [22,23].

Interestingly, diabetes mellitus, traditionally regarded as a major risk factor for CIN, was not significantly associated with AKI in our cohort. This observation is echoed by recent analyses indicating that in patients with preserved baseline renal function and prompt intervention, diabetes may not independently predict AKI [24]. This may reflect effective peri-procedural management and confounding by other stronger predictors such as hemodynamic status and contrast load. Nonetheless, other studies continue to report diabetes as a significant risk factor, particularly in those with pre-existing nephropathy, highlighting the ongoing controversy [25].

Symptom-to-door time was also associated with AKI, suggesting that delayed reperfusion not only compromises myocardial salvage but also predisposes kidneys to ischemia-reperfusion injury [10]. Early intervention thus remains critical to protect both cardiac and renal function. This concept is supported by data demonstrating that prolonged ischemic times increase the risk of AKI and worsen outcomes [11].

Limitations

There are multiple limitations to this study. The results might not be generalizable due to the single-center design and small sample size. Serum creatinine and eGFR alone were used to evaluate baseline renal function; tubular damage indicators were not used to evaluate long-term renal outcomes. Furthermore, it was not possible to adequately account for possible unmeasured variables such lesion complexity and hydration status.

## Conclusions

The findings of this study support the development of a comprehensive risk stratification model incorporating clinical severity (Killip class), contrast volume, and location of the culprit lesion to predict and mitigate AKI risk in PCI candidates. Future studies should explore the potential nephroprotective effects of pharmacologic agents such as SGLT2is in high-risk populations. These findings emphasize the need for early clinical identification of hemodynamic instability in patients undergoing primary PCI. Timely perfusion optimization and contrast usage control can significantly lower AKI risk in everyday practice. More multicenter trials are needed to validate these indicators and create bedside scoring systems for early AKI risk classification in patients with STEMI.
